# A Comparative Analysis of the Core Proteomes within and among the *Bacillus subtilis* and *Bacillus cereus* Evolutionary Groups Reveals the Patterns of Lineage- and Species-Specific Adaptations

**DOI:** 10.3390/microorganisms10091720

**Published:** 2022-08-26

**Authors:** Marios Nikolaidis, Andrew Hesketh, Dimitris Mossialos, Ioannis Iliopoulos, Stephen G. Oliver, Grigorios D. Amoutzias

**Affiliations:** 1 Bioinformatics Laboratory, Department of Biochemistry and Biotechnology, University of Thessaly, 41500 Larissa, Greece; 2 School of Applied Sciences, University of Brighton, Huxley Building, Lewes Road, Brighton BN2 4GJ, UK; 3 Microbial Biotechnology-Molecular Bacteriology-Virology Laboratory, Department of Biochemistry and Biotechnology, University of Thessaly, 41500 Larissa, Greece; 4 Division of Basic Sciences, University of Crete Medical School, 71110 Heraklion, Greece; 5 Department of Biochemistry, University of Cambridge, Cambridge CB2 1GA, UK

**Keywords:** *Bacillus subtilis*, *Bacillus cereus*, core proteome, accessory proteome, fingerprints, phylogenomics, comparative genomics, species-specific adaptations, sporulation

## Abstract

By integrating phylogenomic and comparative analyses of 1104 high-quality genome sequences, we identify the core proteins and the lineage-specific fingerprint proteins of the various evolutionary clusters (clades/groups/species) of the *Bacillus* genus. As fingerprints, we denote those core proteins of a certain lineage that are present only in that particular lineage and absent in any other *Bacillus* lineage. Thus, these lineage-specific fingerprints are expected to be involved in particular adaptations of that lineage. Intriguingly, with a few notable exceptions, the majority of the *Bacillus* species demonstrate a rather low number of species-specific fingerprints, with the majority of them being of unknown function. Therefore, species-specific adaptations are mostly attributed to highly unstable (in evolutionary terms) accessory proteomes and possibly to changes at the gene regulation level. A series of comparative analyses consistently demonstrated that the progenitor of the Cereus Clade underwent an extensive genomic expansion of chromosomal protein-coding genes. In addition, the majority (76–82%) of the *B. subtilis* proteins that are essential or play a significant role in sporulation have close homologs in most species of both the Subtilis and the Cereus Clades. Finally, the identification of lineage-specific fingerprints by this study may allow for the future development of highly specific vaccines, therapeutic molecules, or rapid and low-cost molecular tests for species identification.

## 1. Introduction

*Bacillus* are rod-shaped, Gram-positive aerobic (or facultatively anaerobic) bacteria that form endospores and colonize many diverse habitats [1]. The type species of the genus, *Bacillus subtilis*, was first described by Ehrenberg in 1835 (as *Vibrio subtilis*), whereas the genus was established by Cohn and Koch in 1872 [2]. Members of the genus have been isolated from soil, water, and sediment, as well as from many diverse hosts, such as humans, animals, and plants [3,4], and they act as both human and/or animal pathogens [5]. *Bacillus* species have been exploited as plant growth-promoting factors [6], as pest controllers [7], and as bioreactors for the production of important enzymes, metabolites, antibiotics, food preservatives [8], and probiotics [9]. *Bacillus subtilis* is a very popular model organism for studying Gram-positive bacteria and sporulation as a developmental pathway [10]; it is also emerging as a synthetic biology “chassis” [11]. Moreover, many other *Bacillus* species are also focal points of research, including *B. cereus* (a cause of foodborne illnesses)*, B. thuringiensis* (a pest control agent), and *B. anthracis* (a lethal pathogen of livestock and humans).

Taxonomically, the *Bacillus* genus belongs to the *Firmicutes* phylum and includes more than 104 species that display high diversity (https://lpsn.dsmz.de/genus/bacillus, accessed on 22 August 2022) [4,12,13]. Phylogenetic analyses based on 16S rRNA and on Multi-Locus Sequence Typing (MLST) have studied the evolution and taxonomy of this genus [14]. However, the advent of low-cost whole-genome sequencing technologies has played a critical role in resolving evolutionary relationships at an even finer scale (even at the species or strain level) [15]. This new phylogenomic approach utilizes the phylogenetic signal from hundreds or even thousands of orthologous genes/proteins [16,17,18]. Furthermore, phylogenomics is considered robust against phenomena, such as Horizontal Gene Transfer, that may scramble the evolutionary signal of certain gene families but not the majority of them [19,20,21]. The phylogenomic approach relies on the pangenome concept, where genes and proteins are characterized as core, dispensable/accessory or strain specific, based on their evolutionary/phylogenetic distribution [22,23]. As more genomes become available, the concept of core genes/proteins needs to become more relaxed (i.e., presence in 95% of the analyzed strains) so as to account for sequencing errors, among other things, [24]. In addition, comparisons of average nucleotide identity (ANI) between whole-genome sequences have been utilized in this new genomic era for delimiting species boundaries, usually with an implemented cut-off value of 94–96% identity [25,26].

The importance of the *Bacillus* genus has led to the sequencing of almost 5800 genomes (source: NCBI Assembly; Bethesda, MA, USA, May 2022), with at least 20% of them being annotated as whole-genome or chromosome-level assemblies (high quality). Thus, several recent phylogenomic studies have utilized the wealth of these genomic data to delineate, with much higher accuracy and confidence, the major and minor *Bacillus* lineages and their evolutionary relationships [3,27,28,29,30,31,32,33,34,35,36]. For example, [3] analyzed 79 representative *Bacillus* genomes and identified 196 core genes that were utilized for phylogenomic analyses. They identified 9 well-supported clades within the genus, with the *B. cereus* and *B. subtilis* clades being the most prominent. A recent phylogenomic study based on 352 genomes proposed that the genus should include the two major clades of *B. subtilis* and *B. cereus* and some additional *Bacillus* species, whereas several other previously classified *Bacillus* species should be re-assigned to new genera [4]. A later study of 303 genomes proposed that the *Bacillus* genus should only include the two major clades (termed the Subtilis Clade and Cereus Clade), whereas all the other previously classified *Bacillus* species should become new genera [37]. Based strictly on phylogenomics, the Cereus Clade should also be a distinct genus; however, it has been decided to retain the *Bacillus* name for health and safety reasons [38,39]. Recently, the NCBI taxonomy adopted some of these conclusions and removed several clades from the *Bacillus* genus.

The goal of this study is to utilize the publicly available complete sequences of genomes/chromosomes of *Bacillus* species to identify the distinct evolutionary lineages at the clade and species level based on phylogenomics and ANI values. We also determine the chromosomally encoded core and the fingerprint proteins of these lineages that characterize/define them at both relaxed and strict stringencies. This should reveal any molecular adaptations at the gene/protein content level. We define those fingerprint proteins that are present in all analyzed members of a lineage/group but are absent in all other analyzed *Bacillus* genomes. Therefore, these fingerprints constitute lineage-specific core proteins. In addition, another category of strict fingerprints will be erected that do not have a close homolog (>50% amino acid identity, over 50% of the protein’s length) in any other *Bacillus* lineage. For a more detailed definition of relaxed and strict fingerprints, see Section 2.4. We applied a similar approach in an analysis of the *Pseudomonas* genus and identified fingerprint proteins of *Pseudomonas aeruginosa* (present in all *P. aeruginosa* members, but absent in all other *Pseudomonas* groups) that are involved in its pathogenicity in humans [40].

## 2. Materials and Methods

### 2.1. Analyzed Genomes

A total of 1154 genomes of the *Bacillus* genus (NCBI taxonomy ID: 1386) were downloaded from the NCBI RefSeq database (latest download in April 2022), whose assembly level was annotated as being a complete genome or chromosome. Next, we filtered out genomes that were from confounding strains (i.e., those that have been artificially manipulated) or had less than 10× genome coverage, more than 1% unknown nucleotides, or many pseudogenes (≥10%) [34]. The final dataset contained 1104 genomes. The goal was to filter out all genomes whose assembly level was of lower quality and would result in a significant underestimation of the core proteomes and the accompanying fingerprints.

### 2.2. Orthology Detection and Phylogenomic Analysis

In order to identify the core proteome of this set of organisms, we implemented a series of Python scripts that we developed for studying the core proteome of the *Pseudomonas* genus [40]. In brief, these scripts rely on best reciprocal BLAST hits between a reference proteome of a defined evolutionary group, and all the other proteomes of that evolutionary group that are under investigation. In this way, a core set of orthologs present in them all was identified. Thus, each evolutionary group has its own reference proteome (see Supplementary Excel File S1, spreadsheet 1). For all reciprocal BLAST hits of the reference proteome against another proteome, the Python script gathers all the best reciprocal BLAST-result percent identities, estimates the mean value and standard deviation and then filters out all hits that have identities two standard deviations below the average value. This approach permits the definition of an adjustable orthology cut-off, depending on the genetic distance of the two genomes/proteomes undergoing reciprocal BLAST, instead of fixed cut-offs of sequence identity/similarity or defined BLAST score ratios, as implemented in many other pangenome analyses [22,41,42,43,44]. Afterwards, multiple alignments for all identified groups of core orthologs are generated with Muscle software [45]; they are concatenated in a super-alignment and then filtered with G-blocks software [46] for removing badly aligned regions (default parameters). A maximum likelihood (ML) phylogenomic tree is generated, using the IQTree2 software [47], which automatically calculates the best-fit model. In our study, tree visualization was performed using Treedyn [48] and iTOL [49].

Species boundary determination was based on Average Nucleotide identity with the FastANI [50] software and MUMmer/pyani software [51]. Functional category assignment of the identified core and fingerprint proteins was based on the EGGNOG database v5 [52] and the KEGG Orthologies with the KAAS tool [53] and COG [54].

### 2.3. Detection of Core Proteomes

A protein is considered to be a member of the core proteome of a certain lineage if it is present in all its members. However, the number of proteomes analyzed seriously affects the number of core proteins; more genomes result in fewer core proteins [40]. Given the imbalanced sequencing of the various lineages, we generated a normalized core proteome for each evolutionary lineage of interest by using a maximum number of only five randomly selected proteomes from that lineage. Such normalized datasets allow for meaningful comparisons between lineages of varying sampling coverage, in terms of numbers of genomes. They, nevertheless, remain within the concept of a soft-core proteome [24]. The list of normalized core proteins for each evolutionary lineage (Clade/group/species) is given in Supplementary Excel File S1, spreadsheet 2. We tested the statistical significance of enrichment of a certain functional category within the normalized core proteins of a certain species using the hypergeometric test. The results are summarized in Supplementary Excel File S1, spreadsheet 3.

We investigated if a normalized core proteome based on five genomes would be equivalent to a soft-core proteome at the 85%, 90%, or 95% level, assuming that many more genomes would be available. We thus performed permutation analyses for four different species (*B. subtilis, B. velezensis, B. anthracis*, and Cereus subclade 2) with more than 100 available genomes each. We randomly sampled twenty times each, genomes of that species for different genome numbers available and estimated how many of its proteins would be present in 85%, 90%, or 95% of the selected genomes. We plotted these permutated soft-cores together with the normalized core based on the five or less genomes for that species. Less than five complete genomes results in an even softer core. As it is evident from Supplementary Figure S1, the normalized core should correspond to a soft core of 85% for *B. velezensis*, whereas for the other three species it corresponds to a soft core of between 90–95%.

### 2.4. Detection of Lineage-Specific Relaxed and Strict Fingerprint Proteins

In order to identify fingerprint proteins of a particular evolutionary lineage, we applied two criteria, one relaxed and the other stringent. Based on our relaxed criteria, the orthologs of this protein (relaxed fingerprint) were present in the five (or less) analyzed members of this particular clade (that were used for the normalized core proteome) and absent in all other *Bacillus* proteomes (that were included in normalized datasets). Based on our more stringent criterion (strict fingerprints), the previously identified relaxed fingerprints should additionally not have any other close homolog in any of the other *Bacillus* proteomes with BLASTP percent identity above 50%, across 50% of the protein’s length. From this point on, we will refer to the fingerprint proteins with two numbers, one for the normalized relaxed fingerprints and the other for the normalized strict fingerprints. The list and table of relaxed/strict fingerprints for each evolutionary lineage (Clade/group/species) together with their functional category is given in Supplementary Excel File S1, spreadsheets 3 and 4.

## 3. Results and Discussion

### 3.1. Phylogenomic Analysis of the Bacillus Genus

**Figure 1:**
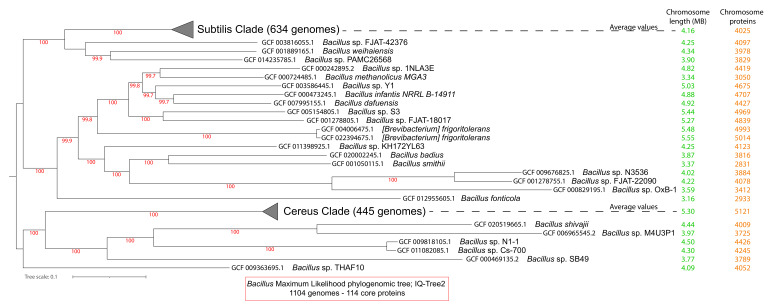
The phylogenomic maximum likelihood tree (IQ-Tree2) of the 1104 *Bacillus* proteomes. The tree was based on 114 core proteins and 20,041 variable sites, using the LG + I + F + G4 model and aLRT. For ease of visualization, the entire Subtilis and Cereus Clades are collapsed. Next to each leaf of the tree, the chromosome size, and the number of all chromosomally encoded proteins are given.

We analyzed all of the 1104 complete proteomes of the *Bacillus* genus, based on the latest NCBI Taxonomy [55]. *B. subtilis strain 168* [10,56] was used as a reference proteome for the whole genus. This was the first Gram-positive bacterium to have its whole genome completely sequenced; moreover, this genome has a high quality annotation [57,58]. In this set of complete proteomes, the most numerous groups were *B. subtilis* strains (194), *B. velezensis* (202), *B. cereus* (131), *B. anthracis* (114)*, B. thuringiensis* (81), and *B. amyloliquefaciens* (58). Our first analysis identified 114 core proteins for the whole *Bacillus* genus (see Supplementary Excel File S1, spreadsheet 2). The multiple alignment of these 114 core proteins contained 20,041 variable sites (after G-blocks filtering) that were used to generate a maximum likelihood phylogenomic tree in IQ-Tree2 (LG + I + F + G4 model-aLRT) [47] (see Figure 1).

We identified two major clades that are also focal points of research in this genus: one that includes *B. subtilis* and many other species and is now referred to in the literature as the Subtilis Clade; and another that includes *B. cereus* and many other species and is now referred to in the literature as the Cereus Clade [37].

### 3.2. Phylogenomic Analysis of the Subtilis Clade

**Figure 2:**
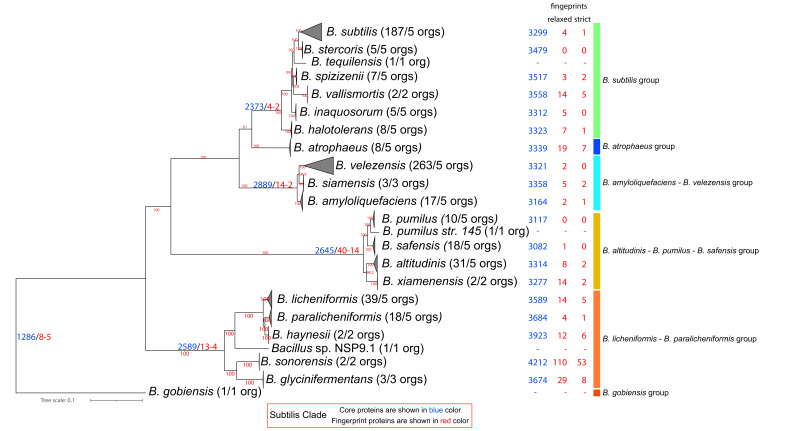
Phylogenomic ML tree (IQ-Tree2-Q.Plant + I + F + G4-aLRT) of the Subtilis Clade based on 457 core protein-orthologous groups from 634 proteomes. For ease of visualization, certain evolutionary clusters have been collapsed. The full tree is available as Supplementary Figure S2. Next to the species name, in parentheses, is the number of complete genomes that are available and, on their right, is the number of genomes used in the normalized dataset. Further to the right of the species names and at the common ancestor of a lineage, with blue and red colors we denote the number of core and relaxed/strict fingerprint proteins for each lineage (based on the normalized dataset).

The Subtilis Clade [1,31,35,37] includes six major groups and 23 species (see Figure 2 and Supplementary Figure S2, for a complete tree): (i) *B. subtilis* (7 species), (ii) *B. atrophaeus* (1 species), (iii) *B. amyloliquefaciens—B. velezensis* (3 species), (iv) *B. altitudinis—B. pumilus—B. safensis* (5 species), (v) *B. licheniformis—B. paralicheniformis* (6 species), (vi) *B*. *gobiensis* (1 species). The members of this clade are hard to distinguish from each other based on phenotypic characteristics or the 16S rRNA phylogeny [59]. This clade has also been verified by our analysis and includes 634 genomes (see Figure 2). It is comprised of 1286 core proteins, with only 8/5 of them being Subtilis Clade relaxed/strict fingerprints, meaning that they are found only within this clade and in no other *Bacillus* proteome that we analyzed. Most of them are of unknown function, whereas one of them is involved in energy production and conversion and another is involved in nucleotide transport and metabolism.

### 3.3. Phylogenomic Analysis of the Cereus Clade

This large phylogenetic clade is organized into three major groups or else subclades [27,28,29,30,60,61,62,63,64,65]. It consists of approximately 30 evolutionary clusters, representing 11 known species and 19–20 putative novel species [29,61]. They are mostly soil bacteria, with some of them being opportunistic pathogens and some being recently emerged pathogens in humans and other organisms [65]. Accordingly, they have been characterized as “hopeful monsters” that can be transformed into pathogens, under the right conditions and circumstances [27,66]. Thus, insights into their evolution are important for understanding basic mechanisms of pathogen emergence. The type species of this large clade is *B. cereus,* a common soil bacterium that is frequently involved in food poisoning [67]. It has also been implicated in skin infection, pneumonia, bacteremia, and meningitis (mostly in immunocompromised individuals) [30]. Its pathogenicity has been attributed to an emetic toxin (cereulide), to enterotoxins/hemolysins, phospholipases, and proteases that function as tissue-destructive exoenzymes. Another prominent member of this clade, *B. thuringiensis,* is an insect pathogen that is characterized by the production of parasporal crystals that contain the insect toxins cry, cyt, and vip, encoded by plasmids [67]. If these plasmids are lost, then *B. thuringiensis* cannot be distinguished from *B. cereus.* Accordingly, *B. thuringiensis* has also been reported as an opportunistic human pathogen [67]. *B. anthracis* is another prominent member of this clade; it was identified by Koch and Pasteur as the etiological agent of anthrax and is pathogenic for both humans and herbivores [67]. Its pathogenicity is mostly attributed to two large plasmids, pXO1 (that encodes three toxin peptides) and pXO2 (that produces the poly-γ-d-glutamic acid antiphagocytic capsule via the capBCADE operon) [68]. The phylogenetic distribution of the pathogenic plasmids and genes has shown that a classification that is mostly based on phenotype and virulence is improper [61,62]. A consistently observed scattered phylogenetic distribution of *B. cereus, B. thuringiensis*, and *B. anthracis* has been the focus of many previous studies. Several studies have proposed that *B. cereus, B. thuringiensis*, and *B. anthracis* should be treated as one species, based on high levels of chromosome synteny and protein similarity [69,70]. The components that differentiate them are mostly attributed to the plasticity of the accessory genomes, with plasmids playing a key role [71]. However, adaptive mutations, recombination events, and re-organization of the gene regulatory network also contribute to this phenotypic heterogeneity. In addition, the impact of positive selection on the core genome shapes the evolution of this lineage [72].

Early comparative analyses of two representative genomes from subclades 1 and 2 vs. *B. subtilis* revealed an under-representation of genes for the degradation of carbohydrate polymers, an abundance of genes encoding proteolytic enzymes, peptide and amino-acid transporters, and a variety of amino-acid degradation pathways [73,74]. Thus, they and others [75] supported the view that the common ancestor of subclades 1 and 2 inhabited the intestine of insects as an opportunistic pathogen, instead of being a benign soil bacterium.

**Figure 3:**
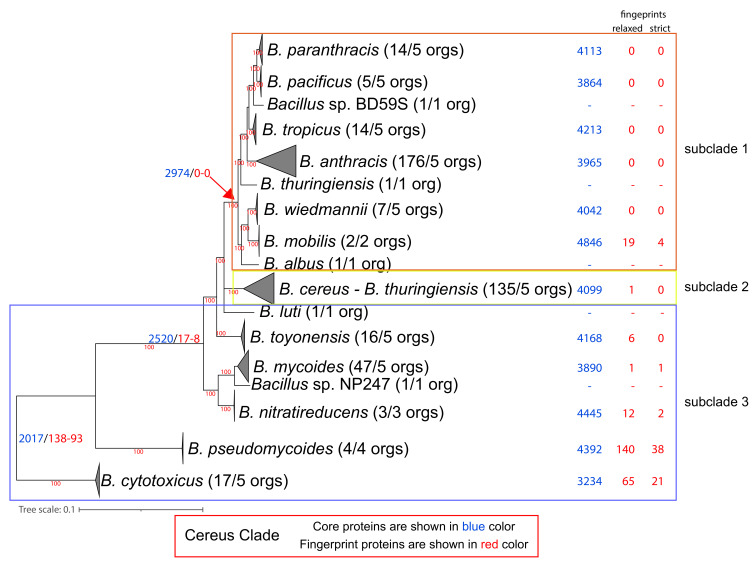
Phylogenomic ML tree (QTree2-Q.Plant + I + F + G4-aLRT) of the Cereus Clade, based on 812 core protein-orthologous groups from 445 proteomes. For ease of visualization, certain evolutionary clusters have been collapsed. The full tree is available as Supplementary Figure S3. Next to the species name, in parentheses, is the number of complete genomes that are available and, on their right, is the number of genomes used in the normalized dataset. Further to the right of the species names and at the common ancestor of a lineage, we denote with blue and red colors the number of core and (relaxed/strict) fingerprint proteins for each lineage (based on the normalized dataset).

Based on our phylogenomic analyses (see Figure 3 and Supplementary Figure S3), we partitioned the Clade into 3 major evolutionary groups or else subclades in accordance with previous evolutionary analyses [27,28,29,30,60,61,62,63,64,65]. Subclade 1 includes 9 species, such as *B. anthracis*; moreover, several *B. cereus* and *B. thuringiensis* strains are also within this subclade. Of note, the *B. anthracis* species (based on the phylogenomic tree and ANI values) includes the clonal lineage as well as several *B. anthracis* Biovars and several strains annotated as *B. cereus* and *B. thuringiensis*. Subclade 2 is organized as a single species (based on the phylogenomic tree and the ANI values) and includes most *B. cereus* (with the reference strain) and *B. thuringiensis* strains. Subclades 1 and 2 are two monophyletic sister groups, whereas subclade 3 is basal and paraphyletic, consisting of seven species. Of note, a few strains annotated as *B. cereus* and *B. thuringiensis* are also found within subclade 3.

Our analysis identified 2017 normalized core proteins for the entire Cereus Clade. We also identified 138/93 (relaxed/strict) fingerprints for the Cereus Clade (as an entire lineage), meaning that these fingerprints are found only within the Cereus clade and in no other *Bacillus* proteome that we analyzed. Notably, the entire Subtilis Clade has 1286 normalized core proteins and only 8/5 (relaxed/strict) fingerprints. This is a strong indication that the common ancestor of the Cereus Clade underwent an extensive series of genomic adaptations in terms of gene content that were most probably shaped by its lifestyle. Alternatively, the common ancestor of the Subtilis Clade could have undergone extensive gene losses. However, the chromosome size as well as the number of chromosomally encoded proteins in the other *Bacillus* species (excluding the Subtilis and Cereus Clades) are very similar to those of the species in the Subtilis Clade (no statistically significant difference) and significantly smaller than the species of the Cereus Clade (*t*-test *p*-value < 0.05). This is a strong indication that the ancestor of the Cereus Clade underwent extensive genomic expansion, rather than the Subtilis Clade experiencing a major loss of genes.

The vast majority of Cereus Clade fingerprints (102/86) are of unknown function. Nevertheless, the other three most numerous functional categories are amino acid transport and metabolism (8/1), cell wall/membrane/envelope biogenesis (5/3), and transcription (4/0).

### 3.4. The Core Proteome and the General Genomic Characteristics of the Genus and Its Species

The general genomic characteristics, the core proteome and the fingerprints of the various lineages, are summarized in Table 1. In addition, the specific normalized core and fingerprint proteins of each lineage and their annotations are available in Supplementary Excel File S1, spreadsheets 2, 3, 4, and 5.

We calculated, using the hypergeometric test, the statistical significance of the fold enrichment/depletion of certain functional categories in the core proteomes of 31 species from the Subtilis and Cereus Clades (see Supplementary Excel File S1, spreadsheet 3). Consistently, in all 31 species, the category of unknown function is significantly depleted, as might be expected. Although their absolute numbers are substantial, the proportion of proteins of unknown function is low. This contrasts with the situation in eukaryotes, where 75% of the proteins of unknown function encoded by the genome of the fission yeast, *Schizosaccharomyces pombe*, are conserved in other fungi and fully 23% are also found in humans [76]. Furthermore, the functional categories of nucleotide transport and metabolism (F); translation, ribosomal structure, and biogenesis (J); energy production and conversion (C); inorganic ion transport and metabolism (P); amino acid transport and metabolism (E); and coenzyme transport and metabolism (H) are consistently enriched in the vast majority (28–30/31) of the species. A recent pangenome analysis focused on 238 strains (ranging between 20–58 per species) of 5 *Bacillus* species and identified their core genes (indicated in the parentheses), namely those of *B. amyloliquefaciens* (2870), *B. subtilis* (1022), *B. anthracis* (3972), *B. cereus* (1656) and *B. thuringiensis* (2299) [77]. For every species, the core gene-set was consistently enriched for functions related to energy production and conversion (C); amino acid transport and metabolism (E); coenzyme transport and metabolism (H); and inorganic ion transport and metabolism (P). Thus, independent studies that utilize different approaches and datasets consistently observe enrichment of the same functional categories.

We also observed that, at the species level, the 31 species of the Subtilis and Cereus Clades had a total of 497 and 162 relaxed and strict fingerprints. Barring three outlier species with very high numbers of fingerprints (*B. sonoresnsis, B. pseudomycoides*, and *B. cytotoxicus*), the average number of relaxed and strict fingerprints for the other species were 7 and 2, respectively. The vast majority of relaxed fingerprints (411/497—83%) were of unknown function, whereas the second and third largest functional categories were cell wall/membrane/envelope biogenesis (M: 16/497—3%) and amino acid transport and metabolism (E: 12/497—2%). Notably, 158/162 (98%) of the species-level strict fingerprints were of unknown function.

We compared several genomic characteristics among the species belonging to the Cereus and Subtilis Clades (see Table 1). Interestingly, we observed that (i) the length of the chromosome was on average 28% higher (5.3 vs. 4.13 Mbp; *t*-test *p*-value < 0.05) in the species of the Cereus Clade; (ii) the number of chromosomally encoded proteins was on average 28% higher (5124 vs. 3989; *t*-test *p*-value < 0.05) in the species of the Cereus Clade; (iii) the number of core proteins was on average 19% higher (4106 vs. 3450; *t*-test *p*-value < 0.05) in the species of the Cereus Clade; and (iv) the number of accessory proteins was on average 89% higher (1018 vs. 539; *t*-test *p*-value < 0.05) in the species of the Cereus Clade. However, when we tested for differences at the level of relaxed and strict fingerprint proteins, no statistically significant difference was observed. Nevertheless, these findings are compatible with our previous findings (see Section 3.3.) that the Cereus Clade has been through an expansion of its genome.

**Figure 4:**
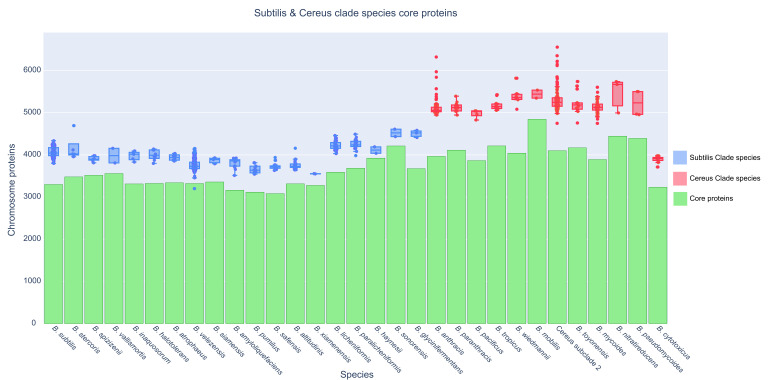
Boxplot of the total number of proteins (y-axis) for every available strain (dot—each genome) of a species (x-axis) and its normalized core proteome (green bar).

For every species of the Subtilis and Cereus Clades, we also plotted the total number of chromosomally encoded proteins per strain (for all available strains with complete genomes) together with the normalized core proteome of that species (see Figure 4). Thus, the extent of variability of the accessory proteome for every species is visualized. Notably, the *B. anthracis* species (including the Anthracis clonal lineage and several other strains—see Supplementary Figure S2), the Cereus subclade 2 species, and the *B. mycoides* species demonstrate an elevated variability in terms of chromosomally encoded accessory proteins.

### 3.5. The Phylogenetic Distribution Profile of the Core and Accessory Protein Components of the Subtilis and Cereus Clades

**Figure 5:**
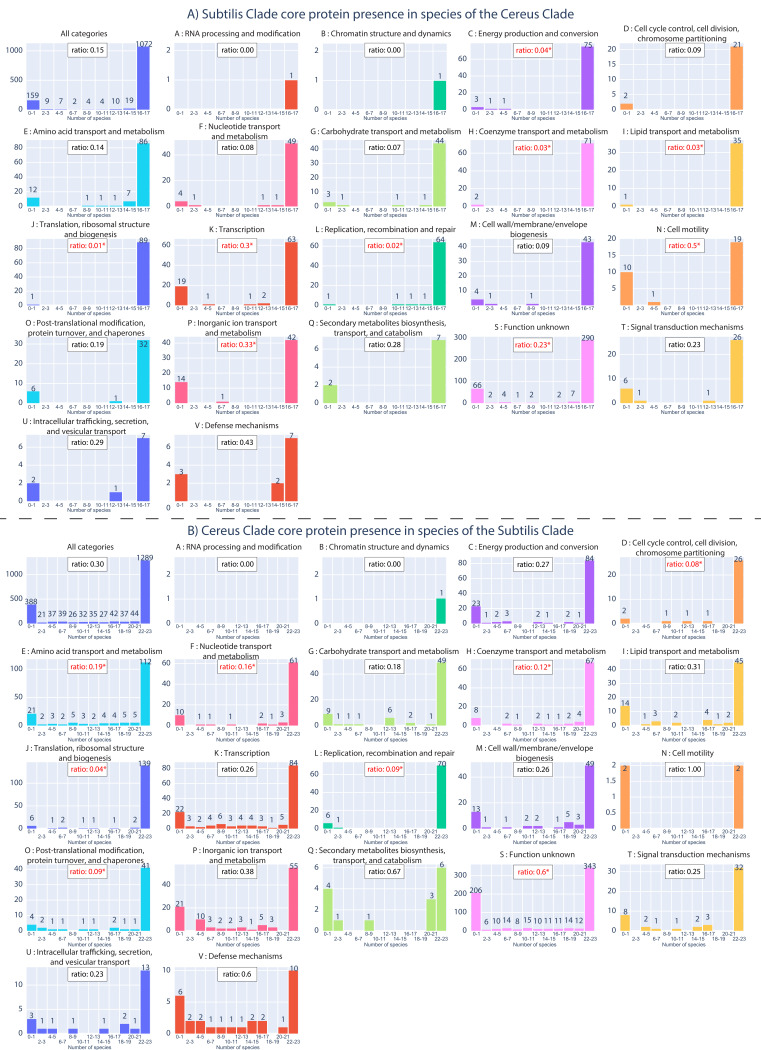
(**A**) The phylogenetic distribution of core proteins of the Subtilis Clade in the species of the Cereus Clade. (**B**) The phylogenetic distribution of core proteins of the Cereus Clade in the species of the Subtilis Clade. The bins on the x-axis correspond to the number of species (in the other Clade), while the y-axis corresponds to the absolute number of core proteins (for that bin). For example, the first graph of Figure 5A shows that 1072 of the core proteins of the Subtilis Clade are also present in 16–17 species of the Cereus Clade. The ratio of the low-presence to high presence bin is shown in the box at the top of the graph. Stars identify any ratio whose difference from the background (in all categories) is statistically significant (based on the hypergeometric test; *p*-value < 0.05).

We investigated what proportion of core proteins of the Subtilis Clade (as an entire lineage) were also present (and how often) in the various species of the Cereus Clade and vice versa (see Supplementary Excel File S1, spreadsheets 6 and 7). In this way, it is possible to understand the similarities of the core genomic components of these two Clades, and determine where they diverge from each other. The results are summarized in Figure 5, for all the core proteins and for each functional category of the core proteins, separately. Overall, the vast majority (1072/1286–83%) of the Subtilis Clade core proteins have a presence in most species (16 or 17 species) of the Cereus Clade; the second largest bin consists of 159 (12%) Subtilis Clade core proteins that have a very low presence (in 0 or 1 species) in the Cereus Clade. This unbalanced bimodal distribution or even unimodal distribution in favor of presence in most species is observed for all individual functional categories as well. Next, we calculated the ratio of the low-presence bin (0–1 species) to the high presence bin (16–17 species) for the entire core proteins (background) and for each functional category separately. In addition, we performed a hypergeometric test to identify any categories that have a significantly different ratio of low-presence core proteins compared to the background (all core proteins). The highest and statistically significant ratio was observed for proteins of unknown function. However, it is noteworthy that Subtilis Clade core proteins belonging to functional categories, such as secondary metabolism, signal transduction, intracellular trafficking/secretion, and defense mechanisms, also have a very low presence in the species of the Cereus Clade, though this is not statistically significant. Therefore, these categories of core proteins may be involved in fundamental adaptations that differentiate the Subtilis Clade from the Cereus Clade.

Analysis of the equivalent phylogenetic distribution profiles of the Cereus Clade core proteins also demonstrated a bimodal-like pattern; the majority of them (1289/2017–64%) have a high presence (22–23) in most species of the Subtilis Clade, whereas 388 (19%) of them have a very low presence (0–1) in the species of the Subtilis Clade. Interestingly, the ratio (0.3) of low/high presence is significantly higher in the Cereus Clade, compared to the equivalent ratio (0.15) in the Subtilis Clade. This is another clear indication that the Cereus Clade is much more specialized in terms of the proteins it encodes, compared to the Subtilis Clade. For the individual functional categories, the highest and statistically significant ratio (0.6) was once again observed for unknown function. Cereus Clade core proteins that belong to functional categories, such as secondary metabolism and defense mechanisms, also have a very low presence in the species of the Subtilis Clade, though this is not statistically significant. Therefore, these core proteins may be involved in fundamental adaptations that differentiate the Cereus Clade from the Subtilis Clade.

We performed a similar analysis for the accessory proteins (i.e., not members of the core set) of each species (we used the reference strain) from one of the two Clades, that have a very low presence (in 0–1 species) in the other Clade. We observed that accessory proteins from species of the Subtilis Clade that had a very low presence in the Cereus Clade were consistently enriched for the category of unknown function (see Supplementary Excel File S1, spreadsheet 8). The same (consistent enrichment of unknown function) applied for accessory proteins from species of the Cereus Clade that had a very low presence in the Subtilis Clade.

### 3.6. The Phylogenetic Distribution Profile of Sporulation and Essential Proteins of the Model Organism Bacillus Subtilis

**Figure 6:**
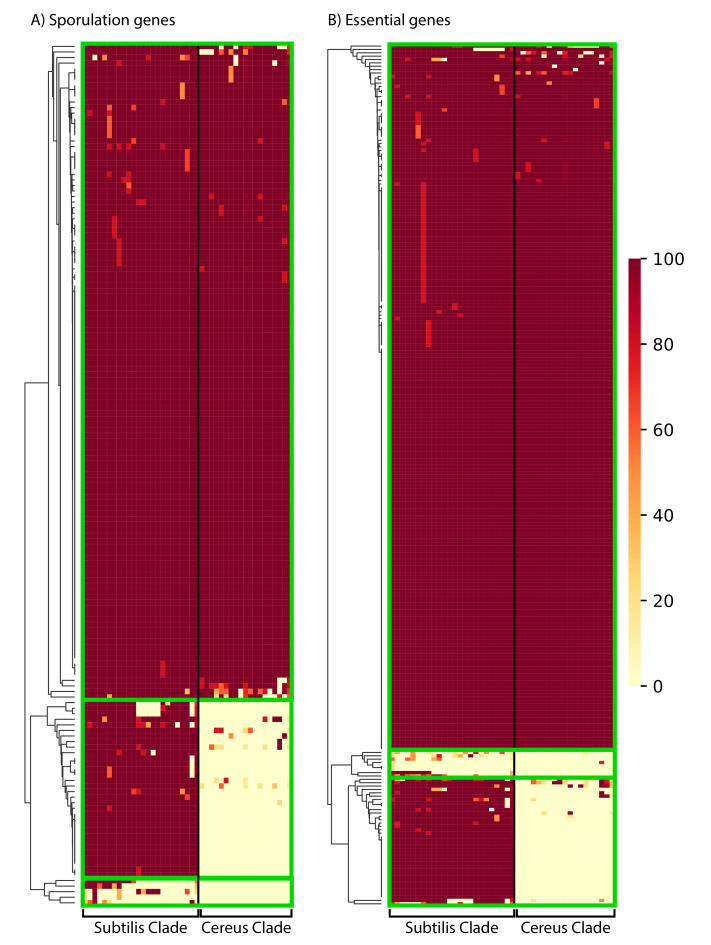
The phylogenetic distribution pattern of: (**A**) 155 *B. subtilis* proteins important for sporulation; (**B**) 256 proteins that are essential in *B. subtilis*. Presence of a close homolog in a given species of the Subtilis and Cereus Clades was determined based on 50% amino acid identity over 50% of the protein’s length. The clustering of proteins (based on their distribution) was performed with the average Euclidean distance, within the seaborn.clustermap python package. A more detailed view of the cluster-heatmaps (including the individual gene names and species) is available in Supplementary Figures S4 and S5. Each row corresponds to a gene and each column corresponds to a certain species. The color in the heatmap corresponds to the % presence (how many strains of the species) of that gene in that certain species.

A major characteristic of Bacilli is their ability to form very resistant spores under harsh conditions [10,78,79]. Accordingly, *B. subtilis* has been widely used as a model organism for understanding bacterial developmental biology [80,81,82,83,84,85]. Although a large number of genes are involved in sporulation [80,81,82,86], a study exploiting a transposon mutagenesis screen identified 155 protein-coding genes whose disruption showed sporulation defects [87]. Thus, we investigated the phylogenetic distribution profile (presence of a homolog with 50% aa identity over 50% of the protein’s length) of each of these 155 important proteins in the various species of the Subtilis and Cereus Clades (see Figure 6, Supplementary Figure S4, and Supplementary Excel File S1, spreadsheet 9 for details—gene names and distribution patterns). The vast majority (118/155–76%) of these important sporulation proteins have a close homolog in the majority of species, in both the Subtilis and the Cereus Clades. This is a clear indication that most of the crucial genetic components of sporulation are highly conserved across the entire genus. However, we also identified a significant number of key sporulation proteins with very limited phylogenetic distribution, or even absence, from a given lineage. For example, 33 of the 155 sporulation proteins (21%) have a close homolog in the majority of species within the Subtilis Clade, but not in the majority of species within the Cereus Clade. Such sporulation proteins may either be missing from the species of the Cereus Clade, or they have undergone rapid divergence and did not pass our identity criteria. Furthermore, an even smaller number (4/155–3%) of these important sporulation proteins have a very limited presence, even within the Subtilis Clade. Most probably, these proteins are missing from the other species of the Subtilis and Cereus Clades, because there would not have been sufficient evolutionary time to allow their divergence in close relatives to a level below the threshold (50% identity).

A very similar phylogenetic distribution pattern was observed for the 256 proteins of *B. subtilis 168* model strain that have been experimentally determined to be essential (see Figure 6, Supplementary Figure S5, and Supplementary Excel File S1, spreadsheet 10 for details—gene names, and distribution patterns). This list of essential genes was based on the data in Subtiwiki [86]. Again, the majority of these proteins (210/256–82%) have a wide distribution (presence of a homolog with 50% aa identity over 50% of the protein’s length) in the majority of species of both the Subtilis and Cereus Clades. A rather limited number (39/256–15%) have no close homologs in most species of the Cereus Clade. Such *B. subtilis* essential proteins may either be missing from the species of the Cereus Clade, or they have undergone rapid divergence and did not pass our identity criteria. Furthermore, a very small number (7/256–3%) have a very limited distribution even within the Subtilis Clade. Most probably, these proteins are missing from the other species of the Subtilis and Cereus Clade because they would not have sufficient evolutionary time to allow their divergence in close relatives to a level below the threshold (50% identity).

## 4. Conclusions

All of the analyses in our study clearly and consistently demonstrate that the Cereus Clade is considerably more complex and diverse, in terms of its content of chromosomally encoded proteins, compared to the Subtilis Clade. A very significant proportion of proteins that distinguish the Cereus Clade are still of an unknown function, whereas other functional categories are related to secondary metabolism/transport/catabolism and defense mechanisms. *B. subtilis* is the well-established model organism for Bacilli and even for Gram-positive bacteria. However, several of the important components of the human/animal pathogens of the Cereus Clade are not present in *B. subtilis*. Therefore, this study demonstrates the strengths and the limitations of *B. subtilis* as a model organism for certain functions, including pathogenesis. A remarkable observation was that many species in both the Subtilis and the Cereus Clades have very low numbers of fingerprint proteins, with a few notable exceptions. Thus, it emerges that many of these species are much more homogeneous in terms of core protein content than was originally thought and that the species concept is much more relaxed; it is probably based on phenotypic characteristics whose molecular background is very unstable/dynamic. It is also plausible that many species adaptations could be related to gene-regulation, which would not be detected by our approach. Our observations concerning the phylogeny and fingerprints of the various species within the Cereus Clade suggest that subclades 1 and 2, together with several other species from subclade 3, should form one rather homogeneous monophyletic group. In contrast, both *B. pseudomycoides* and *B. cytotoxicus* are so divergent in terms of phylogenomics and fingerprints, that they should form two distinct monophyletic groups within the Clade. Finally, the identification of lineage-specific fingerprints may allow for the future development of highly specific vaccines, therapeutic molecules, or rapid and low-cost molecular tests for species identification.

## Figures and Tables

**Table 1:** The genomic characteristics of the evolutionary lineages and species of the Subtilis and Cereus Clades. The number of core proteins and fingerprints for each clade/group/species are based on the normalized dataset. As normalized, we denote a dataset where the maximum number of genomes used per species is five (or less, if not available). Thus, sampling biases for certain lineages with very high numbers of available genomes are mitigated and the results between different lineages become comparable. The stars indicate species with only one or two available complete genomes.

Taxonomy	Num. Genomes	Avg. Chrom. Length (MB)	Avg. Num of Chrom. Proteins	Core Proteins Normalized	Avg. Num of Accessory Proteins	Relaxed Fingerprints	Strict Fingerprints	Fingerprints (Relaxed/Strict) with Function Unknown
Subtilis Clade	630	4.06	3914	1286	2628	8	5	6/5
*B. subtilis* group	214	4.14	4061	2373	1688	4	2	4/2
*B. subtilis* species	187	4.14	4070	3299	771	4	1	4/1
*B. stercoris* species	5	4.29	4161	3479	682	0	0	0/0
*B. tequilensis* species *	1	4.01	3943	-	-	-	-	-
*B. spizizenii* species	7	4.08	3905	3517	388	3	2	3/2
*B. vallismortis* species *	2	4.12	3983	3558	425	14	5	14/5
*B. inaquosorum* species	5	4.24	3979	3312	667	5	0	5/0
*B. halotolerans* species	8	4.13	3999	3323	676	7	1	6/1
*B. atrophaeus* group	8	4.19	3939	3339	600	19	7	17/6
*B. amyloliquefaciens—B. velezensis* group	283	4.00	3768	2889	879	14	2	12/2
*B. amyloliquefaciens* species	17	3.93	3810	3164	646	2	1	2/1
*B. velezensis* species	263	4.01	3764	3321	443	2	0	2/0
*B. siamensis* species	3	4.12	3870	3358	512	5	2	3/1
*B. pumilus-B. safensis-B. altitudinis* group	61	3.77	3723	2645	1078	40	14	28/14
*B. pumilus* species	10	3.73	3661	3117	544	0	0	0/0
*B. altitudinis species*	31	3.77	3753	3314	439	8	2	8/2
*B. safensis species*	18	3.78	3725	3082	643	1	0	1/0
*B. xiamenensis* species *	2	3.63	3553	3277	276	14	2	13/2
*B. pumilus str. 145* *	1	3.94	3898	-	-	-	-	-
*B. licheniformis—paralicheniformis* group	64	4.36	4252	2589	1663	13	4	7/4
*B. licheniformis* species	39	4.30	4228	3589	639	14	5	12/5
*B. paralicheniformis* species	18	4.42	4248	3684	564	4	1	4/1
*B. haynesii* species *	2	4.28	4115	3923	192	12	6	12/6
*Bacillus* sp. NSP9.1 *	1	4.54	4496	-	-	-	-	-
*B. sonorensis species* *	2	4.72	4522	4212	310	110	53	93/52
*B. glycinifermentans* species	3	4.67	4505	3674	831	29	8	27/8
*B. gobiensis* group	1	4.60	4455	-	-	-	-	-
Cereus Clade	305	5.24	5062	2017	3045	138	93	102/86
Cereus subclade 1	218	5.28	5115	2974	2141	0	0	0/0
*B. paranthracis* species	14	5.28	5134	4113	1021	0	0	0/0
*B. pacificus* species	5	5.13	4984	3864	1120	0	0	0/0
*Bacillus* sp. BD59S *	1	5.28	5168	-	-	-	-	-
*B. tropicus* species	14	5.34	5177	4213	964	0	0	0/0
*B. anthracis* species	176	5.27	5097	3965	1132	0	0	0/0
*B. anthracis clonal* clade	111	5.23	5035	4881	154	45	15	37/15
*B. thuringiensis* *	1	5.33	5198	-	-	-	-	-
*B. wiedmannii species*	7	5.55	5391	4042	1349	0	0	0/0
*B. mobilis* species *	2	5.56	5441	4846	595	19	4	17/4
*B. albus* species *	1	5.30	5101	-	-	-	-	-
Cereus subclade 2	135	5.53	5305	4099	1206	1	0	1/0
Cereus subclades 1 & 2	218	5.28	5115	2881	2234	0	0	0/0
Cereus subclade 3	-	-	-	-	-	-	-	-
*B. luti* species *	1	5.20	4992	-	-	-	-	-
*B. toyonensis* species	16	5.40	5217	4168	1049	6	0	4/0
*B. mycoides* species	47	5.32	5144	3890	1254	1	1	1/1
*Bacillus* sp. NP247 *	1	5.28	5107	-	-	-	-	-
*B. nitratireducens* species	3	5.57	5468	4445	1023	12	2	10/2
*B. pseudomycoides* species	4	5.49	5231	4392	839	140	38	101/37
*B. cytotoxicus* species	17	4.19	3897	3234	663	65	21	51/21
Cereus subclade1&2-mycoides CA	284	5.30	5129	2520	2609	17	8	13/8

## Data Availability

Not applicable.

## Supplementary Materials

The following supporting information can be downloaded at: https://www.mdpi.com/article/10.3390/microorganisms10091720/s1. Figure S1: Saturation curves of soft core proteins at 85%, 90% and 95% depending on the number of genomes sampled; Figure S2: Subtilis clade tree based on 457 core proteins, IQ-Tree2; Figure S3: Cereus Clade tree based on 812 core proteins, IQ-Tree2; Figure S4: *B. subtilis* sporulation gene homologues; Figure S5: *B. subtilis* essential gene homologues. Supplementary Excel File S1: Information on Core and Fingerprint Genes for the various Bacillus lineages.
