# Supplementary material for: A Comparative Analysis of the Core Proteomes within and among the Bacillus subtilis and Bacillus cereus Evolutionary Groups Reveals the Patterns of Lineage- and Species-Specific Adaptations
Source: Microorganisms. 2022 Aug 26;10(9):1720. doi: 10.3390/microorganisms10091720 (PMC9505155; doi:10.3390/microorganisms10091720)

Supplementary Figure S1

Saturation curves of soft core proteins at 85%, 90% and 95% depending on the number of genomes sampled

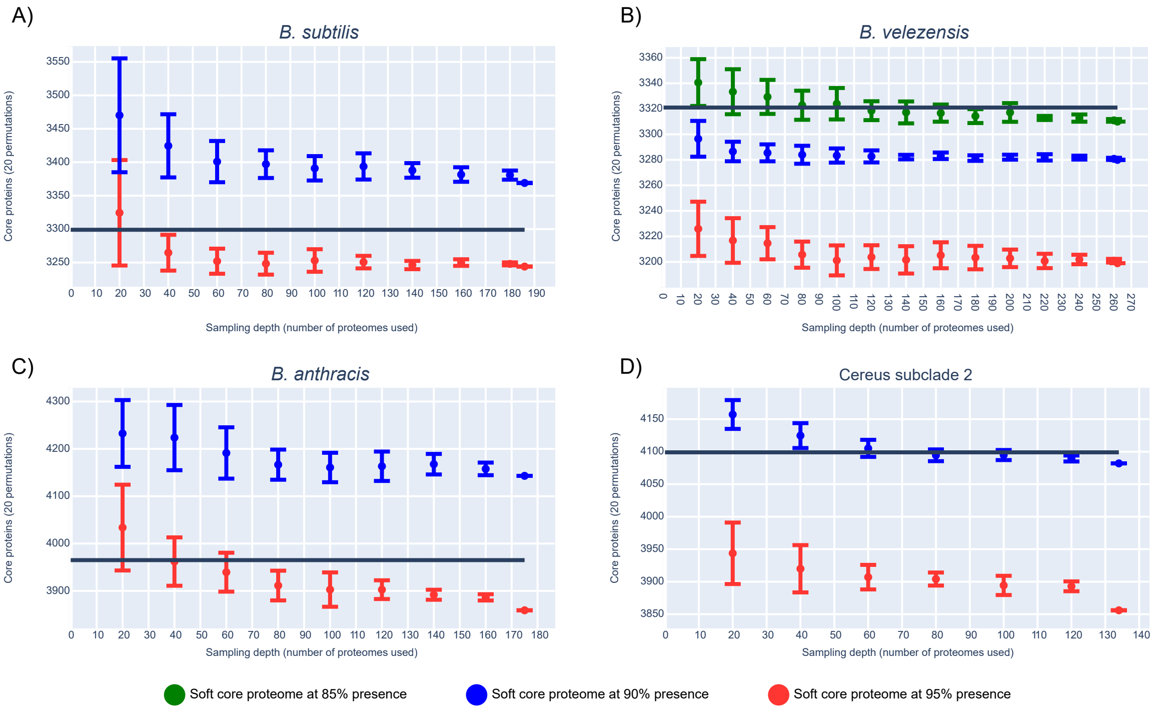

Black bar corresponds to the number of core proteins calculated from the normalized dataset (5 organisms per species)

Supplementary Figure S2: Subtilis Clade tree
Based on 457 core proteins, IQ-Tree2

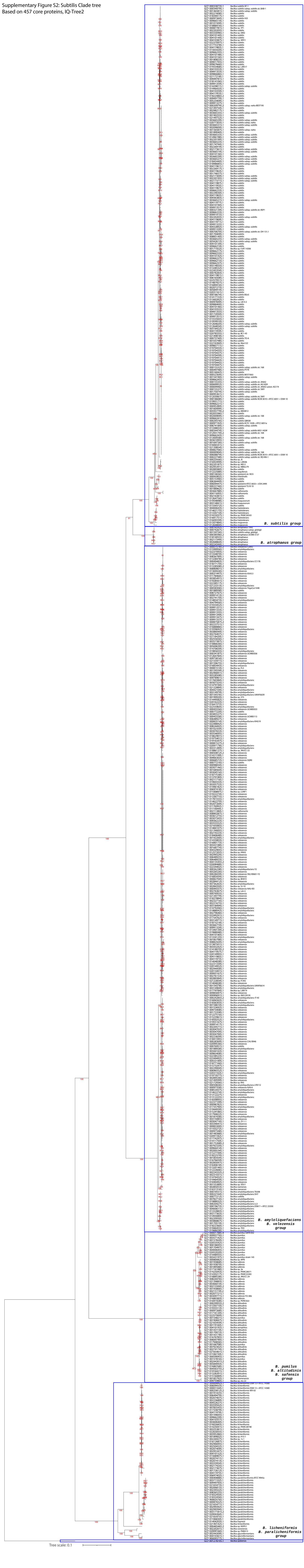

Supplementary Figure S3: Cereus Clade tree
Based on 812 core proteins, IQ-Tree2

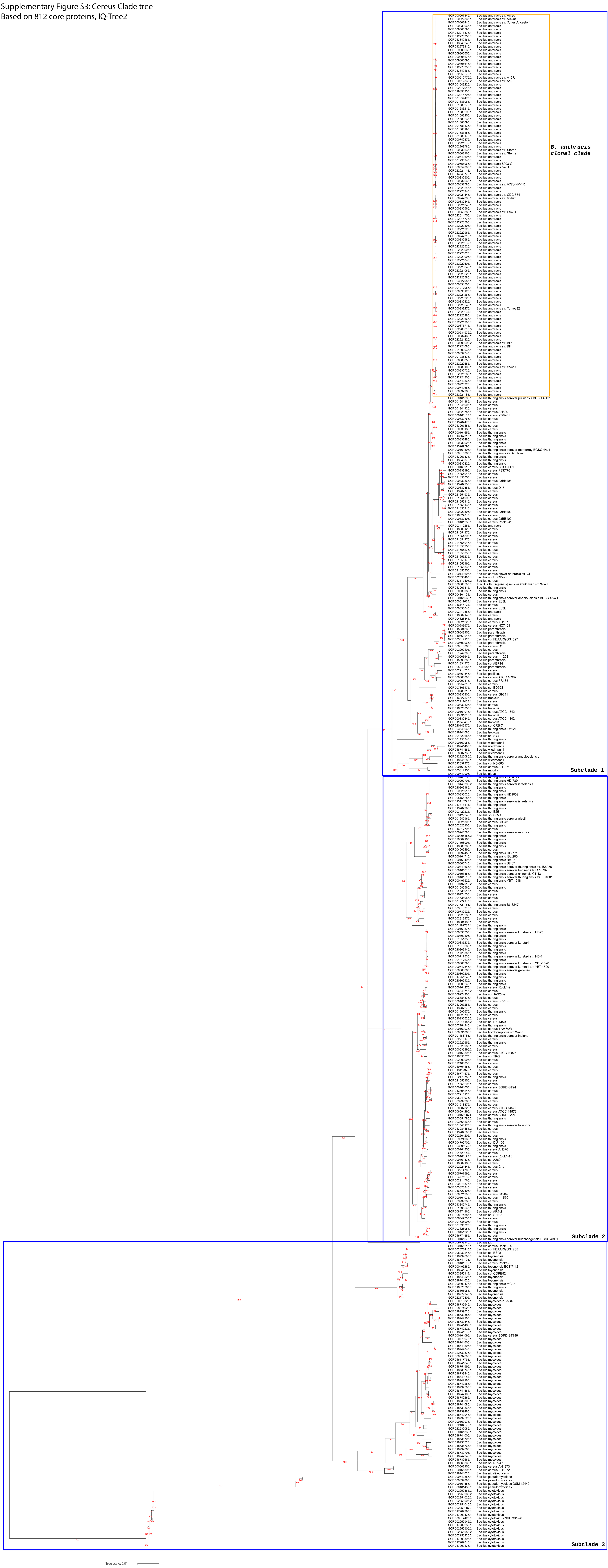

Supplementary Figure S4: Sporulation gene homologues
Presence of homologues with 50% aa identity at 50% protein length
Normalized dataset

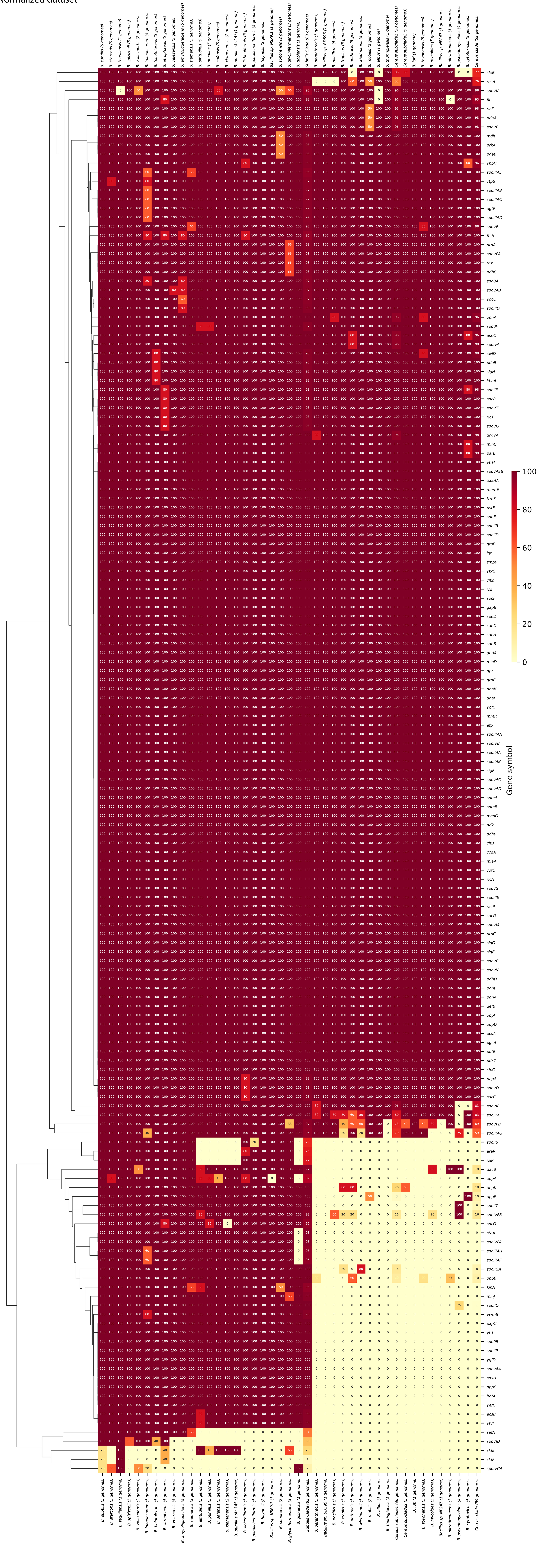

Supplementary Figure S5: Essential gene homologues
Presence of homologues with 50% aa identity a 50% protein length
Normalized dataset

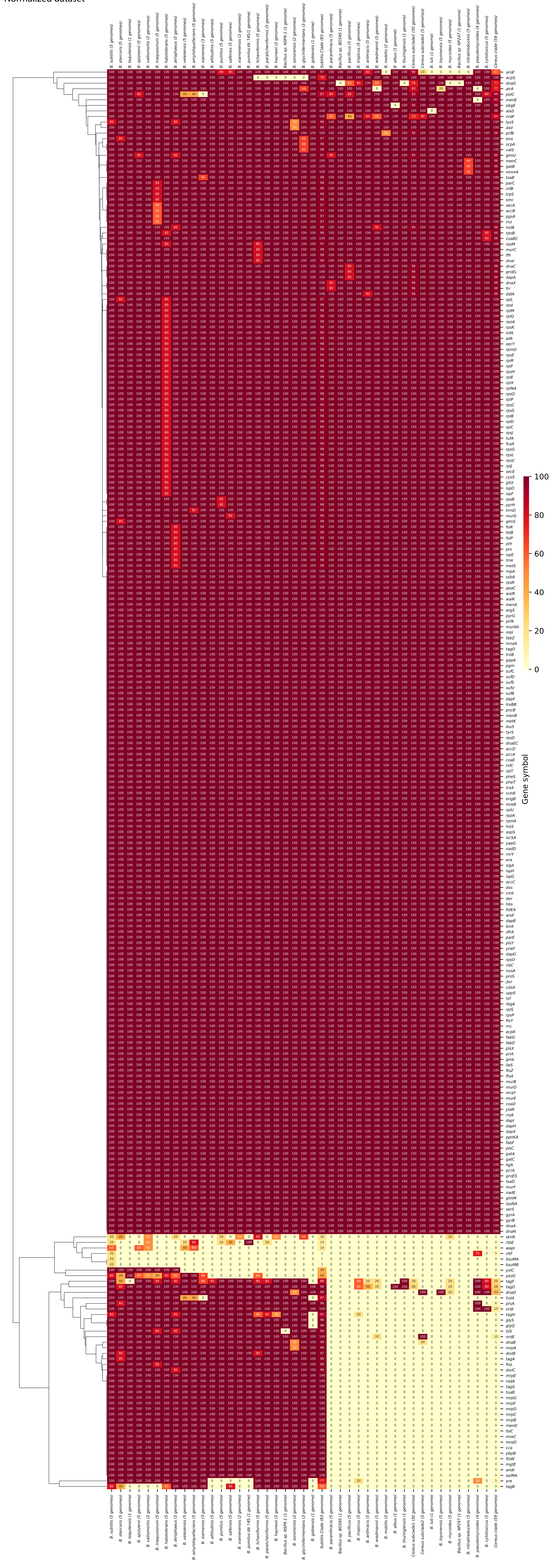

Supplement: Supplementary file 1 [file microorganisms-10-01720-s001.zip › Supplementary_figures_20220530.pdf]
